# Acute Myocardial Infarction: A Comparison of the Risk between Physicians and the General Population

**DOI:** 10.1155/2015/904328

**Published:** 2015-02-23

**Authors:** Yen-ting Chen, Chien-Cheng Huang, Shih-Feng Weng, Chien-Chin Hsu, Jhi-Joung Wang, Hung-Jung Lin, Shih-Bin Su, How-Ran Guo, Chi-Wen Juan

**Affiliations:** ^1^Department of Emergency Medicine, Chi-Mei Medical Center, Tainan, Taiwan; ^2^Department of Environmental and Occupational Health, College of Medicine, National Cheng Kung University, Tainan, Taiwan; ^3^Department of Child Care and Education, Southern Taiwan University of Science and Technology, Tainan, Taiwan; ^4^Department of Emergency Medicine, Kuo General Hospital, Tainan, Taiwan; ^5^Department of Medical Research, Chi-Mei Medical Center, Tainan, Taiwan; ^6^Department of Healthcare Administration and Medical Informatics, Kaohsiung Medical University, Kaohsiung, Taiwan; ^7^Department of Biotechnology, Southern Taiwan University of Science and Technology, Tainan, Taiwan; ^8^Department of Emergency Medicine, Taipei Medical University, Taipei, Taiwan; ^9^Department of Leisure, Recreation and Tourism Management, Southern Taiwan University of Science and Technology, Tainan, Taiwan; ^10^Department of Occupational Medicine, Chi-Mei Medical Center, Tainan, Taiwan; ^11^Department of Medical Research, Chi-Mei Medical Center, Liouying, Tainan, Taiwan; ^12^Department of Occupational and Environmental Medicine, National Cheng Kung University Hospital, Tainan, Taiwan; ^13^Department of Emergency Medicine, Kuang-Tien General Hospital, Taichung, Taiwan; ^14^Department of Nursing, Hungkuang University, Taichung, Taiwan

## Abstract

Physicians in Taiwan have a heavy workload and a stressful workplace, both of which may contribute to cardiovascular disease. However, the risk of acute myocardial infarction (AMI) in physicians is not clear. This population-based cohort study used Taiwan's National Health Insurance Research Database. We identified 28,062 physicians as the case group and randomly selected 84,186 nonmedical staff patients as the control group. We used a conditional logistic regression to compare the AMI risk between physicians and controls. Subgroup analyses of physician specialty, age, gender, comorbidities, area, and hospital level were also done. Physicians have a higher prevalence of HTN (23.59% versus 19.06%, *P* < 0.0001) and hyperlipidemia (21.36% versus 12.93%, *P* < 0.0001) but a lower risk of AMI than did the controls (adjusted odds ratio (AOR): 0.57; 95% confidence interval (CI): 0.46–0.72) after adjusting for DM, HTN, hyperlipidemia, and area. Between medical specialty, age, and area subgroups, differences in the risk for having an AMI were nonsignificant. Medical center physicians had a lower risk (AOR: 0.42; 95% CI: 0.20–0.85) than did local clinic physicians. Taiwan's physicians had higher prevalences of HTN and hyperlipidemia, but a lower risk of AMI than did the general population. Medical center physicians had a lower risk than did local clinic physicians. Physicians are not necessary healthier than the general public, but physicians, especially in medical centers, have a greater awareness of disease and greater access to medical care, which permits timely treatment and may prevent critical conditions such as AMI induced by delayed treatment.

## 1. Introduction

There is a relationship between physician health and quality of care [1]. There are higher risks of medical errors, adverse events, and attentional failures by physicians who frequently work extended shifts [2–4]. The Institute of Medicine reported that every year between 48,000 and 98,000 deaths occur because of iatrogenic errors [4]. Moreover, it is common for physicians to be reluctant to seek health care from their colleagues because they do not want to be embarrassed or are afraid of losing their job or other reasons [1]. Consequently, physicians may tend to work through illnesses and provide patients inappropriate care [1].

In Taiwan, the 1995 launch of the National Health Insurance (NHI) program dramatically changed the nation's health services industry. Universal health insurance gave all citizens equal access to health care [5]. By the end of 2012, almost all (99.85%) of the country's eligible population had enrolled in the NHI [5]. Because of the increased demand for healthcare, Taiwan's physicians may have greater workloads than do physicians in other nations [6]. A study reported that the comparison of mean hours of physicians' work was 51 h/week in US; 46.3 h/week in European Union; 50–70 h/week in New Zealand; and 46–85 h/week in Taiwan [7]. Half of Taiwanese physicians work more than 57 h/week, 34.5% work as many as 65 h/week, and that 10.6% need an average of 21 extra work hours for morning meetings, academic research, and teaching [7]. In addition, the increase of lawsuits for malpractice produces another source of stress for physicians in Taiwan [7]. The number of physicians per 1,000 people in Taiwan was 1.8 in 2012, much less than in Singapore (1.9), Korea (2.1), Japan (2.3), the United States (2.5), and the United Kingdom (2.8) [8–10]. Moreover, the availability of NHI has increased the demand for healthcare in Taiwan. For example, the number of outpatient visits per person increased from 7.89 in 1992 to 15.2 in 2010 [8, 11]. The annual number of outpatient visits per physician in Taiwan increased to 8,600 in 2012, from only 6,621 in 1992, which indicates that workloads and stress levels are increasing for physicians in Taiwan [8, 11].

One study [1] showed that physicians in Taiwan are at higher risks of developing respiratory system and neoplastic diseases compared with other healthcare workers. Compared with the general population, however, their risks of hospitalization were lower for all causes and for seven major disease-specific standardized hospitalization ratios: (i) neoplasms; (ii) endocrine, nutritional, and metabolic disease and immunity disorders; (iii) mental disorders; (iv) circulatory system diseases; (v) respiratory system diseases; (vi) genitourinary system diseases; (vii) and musculoskeletal system and connective tissue diseases. Individual diseases within these seven categories were not provided; therefore, we wanted to investigate acute myocardial infarction (AMI) in Taiwan's physicians, in which a stressful workplace was a significant risk factor [12]. We hypothesized that physicians have a higher risk of AMI than does the general population.

## 2. Methods

### 2.1. Data Sources

Taiwan's NHI Program, a universal healthcare system that covers 99% of the country's population of 23.3 million, has one of the largest and most complete population-based healthcare claims datasets in the world [13]. The NHI Research Database (NHIRD) contains encrypted patient identification numbers, ICD-9-CM (International Classification of Diseases, Ninth Revision, Clinical Modification) codes for clinical diagnoses and procedures, details of prescribed drugs, dates of admission and discharge, and basic sociodemographic information, including gender and date of birth. Information on medical personnel (including physicians and other healthcare providers) is also available and includes date licensed, specialty, work area, hospital level, types of employment, and encrypted identification number, which can be linked to the aforementioned claims data. All the expenses for diabetes mellitus (DM), hypertension (HTN), hyperlipidemia, and AMI are covered by NHI.

### 2.2. Ethics Statement

This study was conducted according to the Declaration of Helsinki and was approved by the Institutional Review Board (IRB) at Chi-Mei Medical Center. The IRB waived the need for informed consents (written and oral) from the patients because the dataset used in this study consists of nationwide, unidentifiable, secondary data released to the public for research. This waiver does not adversely affect the rights and welfare of the patients.

### 2.3. Selection of Cases and Controls

**Figure 1:**
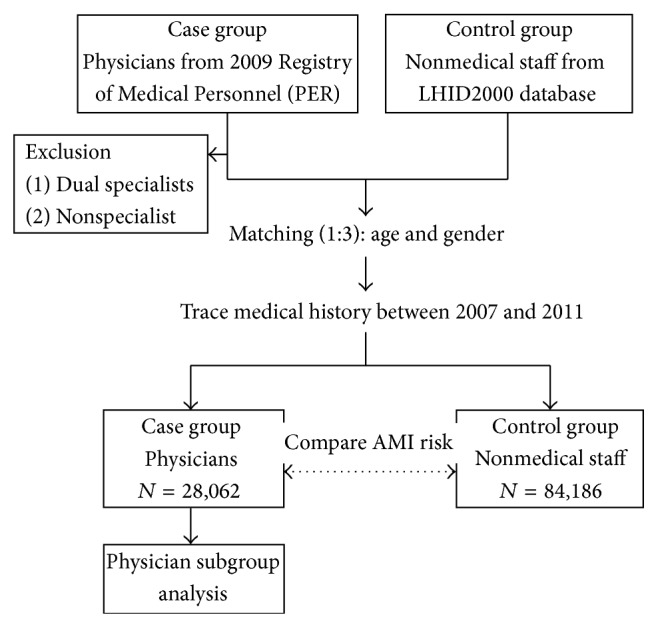
Flow chart for the study. AMI: acute myocardial infarction; LHID: Longitudinal Health Insurance Database.

Data on the physicians were obtained from the Registry of Medical Personnel (PER), which contained all registered medical staff in 2009. We then excluded physicians who were dual specialists (e.g., a physician board certified in internal medicine and emergency medicine) and physicians who were not specialists (i.e., residents) (Figure 1). We excluded dual specialists because of the difficulty involved with assigning them to a specific subgroup for comparison. We excluded residents because their practice time in individual specialties is short. In the control group, we selected three matches (nonmedical staff) per case from the Longitudinal Health Insurance Database 2000 (LHID2000), which contains all claims data of one million (4.34% of the total population) beneficiaries who were randomly selected in 2000 (Figure 1). There are no significant differences in age, gender, or health care costs between the LHID2000 and all NHI enrollees. Controls were matched with cases by age, birth year, and gender (Figure 1).

We linked to the diagnostic codes through the inpatient and ambulatory care claims databases of the NHI. Common comorbidities that might affect the risk of AMI are DM (ICD-9 code 250), HTN (ICD-9 codes 401–405), and hyperlipidemia (ICD-9 codes 272). These three comorbidities were counted if they were diagnosed in 3 or more ambulatory care claims coded 12 months before January 1, 2009, index medical care date.

### 2.4. Exposure Assessment

We compared the risk of AMI between physicians and controls by tracing their medical histories between 2007 and 2011 (Figure 1). AMI was identified using a computerized algorithm that included the ICD-9 code of 410.

### 2.5. Physician Subgroup Analysis

We also analyzed the subgroups of physicians for specialty, age, gender, geographical area, comorbidities, and hospital level (Figure 1). The specialists who practice in emergency and critical care (i.e., internal medicine, surgery, obstetrics and gynecology, pediatrics, and emergency medicine) may have a higher risk of having an AMI because they lead more stressful lives. Therefore, we divided physicians into six subgroups for comparison: internal medicine, surgery, obstetrics and gynecology, pediatrics, emergency medicine, and others (e.g., dermatology and family medicine).

### 2.6. Statistical Analyses

Differences in baseline characteristics and comorbid variables between the two groups were evaluated using Student's *t* test for continuous variables and Pearson *χ* ^2^ tests for categorical variables. We used conditional logistic regression to obtain the odds ratio (OR) of AMI between physicians and controls. Moreover, for the physician subgroup analysis, an unconditional multiple logistical regression was used to explore the risk of AMI in the different specialties. SAS 9.3.1 for Windows (SAS Institute, Cary, NC, USA) was used for all analyses. Significance was set at *P* < 0.05 (two-tailed).

## 3. Results

### 3.1. Baseline Characteristics of Patients

We recruited 28,062 physicians and 84,186 age- and gender-matched controls (Table 1). The median age of the physicians was 46.81 ± 10.75 years, and the proportion of 35- to 49-year-olds was 50.75%. Most physicians were men (85.72%), lived in northern Taiwan (46.86%), and worked in a medical center (43.66%). Comparing with controls, more physicians than controls had HTN (23.59% versus 19.06%, *P* < 0.0001) and hyperlipidemia (21.36% versus 12.93%, *P* < 0.0001), but fewer had DM (8.09% versus 9.51%, *P* < 0.0001). There were 6,745 (24.04%) physicians who specialized in internal medicine, 4,429 (24.04%) in surgery, 3,032 (24.04%) in pediatrics, 2,251 (8.02%,) in obstetrics and gynecology, 552 (1.97%) in emergency medicine, and 11,053 (39.39%) in other areas (Table 1).

### 3.2. Primary Outcome

One hundred and four of the 28,062 physicians (0.37%) and 412 of the 84,186 controls (0.49%) had an AMI between 2007 and 2012 (Table 2). The crude OR was 0.76 (95% CI: 0.61–0.94). After adjusting for DM, HTN, hyperlipidemia, and geographical area, physicians had a significantly lower risk for having an AMI than did the controls (AOR: 0.57; 95% CI: 0.46–0.72) (Table 2).

### 3.3. Subgroup Analyses of Physicians

There were no significant differences in the risk for having an AMI between the subgroups of specialists, age, or geographical area (Table 3). Physicians with chronic comorbidities had a higher risk for having an AMI: HTN (AOR: 7.10; 95% CI: 4.05–12.45); hyperlipidemia (AOR: 2.69; 95% CI: 1.75–4.15); and DM (AOR: 1.83; 95% CI: 1.19–2.81), and physicians working in medical centers (AOR: 0.42; 95% CI: 0.20–0.85) and regional hospitals (AOR: 0.51; 95% CI: 0.28–0.95) had significantly lower risks than did physicians working in local hospitals (AOR: 0.81; 95% CI: 0.48–1.38) and local clinics (AOR: 1.000) (Table 3).

## 4. Discussion

To the best of our knowledge, this is the first study to report the AMI risk in physicians. Using a population-based, cohort study with a large sample, we found that Taiwanese physicians had a higher prevalence of HTN and hyperlipidemia but a lower risk for having an AMI than did the general population. In subgroup analysis, physicians who practiced in specialties related to emergency and critical care medicine did not have a greater risk of having an AMI. Physicians who work in medical centers and regional hospitals had significantly lower risks of having an AMI than did physicians in local hospitals and clinics. Our findings suggest that medical knowledge, higher disease awareness, and easier healthcare access by physicians may help reduce their AMI risk. However, Taiwan's physicians are overworked, and this is still an important issue for physician health and patient safety. That also may account for the higher HTN and hyperlipidemia in physicians. Despite our findings, we cannot conclude that physician health is not significantly affected by their excessive and often onerous workloads.

Physicians are not necessary healthier; however, because of their greater awareness of the symptoms of disease and easier access to medical care, they can often undergo timely treatment if they develop an acute and potentially critical illness [14]. Being a physician is frequently considered to be a profession with high levels of stress [15, 16] and psychological distress [17, 18]. Stressful jobs are associated with adverse effects of diseases of the circulatory system, which trigger the release of catecholamines and high blood pressure, both of which are known risk factors for cardiovascular diseases [19].

The prevalence of chronic comorbidities in physicians is likely underestimated: they may be overlooked because of self-medication and embarrassment should the doctor have to assume the role of a patient [20–23]. In spite of the underestimation, however, the present study revealed that the prevalence of HTN and hyperlipidemia was still higher in physicians than in the general population. Because of their greater awareness, physicians are in a position to seek more rapid medical care to prevent catastrophic events like an AMI. A population-based study [14] on severe sepsis in physicians reported that physicians are less likely than controls to develop or die of severe sepsis, because their medical knowledge and easier access to healthcare may help reduce their risk of severe sepsis and associated mortality.

There was no difference in the risk of having an AMI between specialties in this study. We did not find any study about physician AMI in the literature. However, a study in Taiwan also showed that there was no significant difference of hospitalization due to circulatory disease among physician specialties [1]. Physicians working in internal medicine, surgery, obstetrics and gynecology, and emergency medicine related to emergency and critical care need to work rotating night shifts; long, continuous work hours; and on call. They may have more job stress and burnout than do other specialists [7]. Similarly, physicians working in medical centers may have more stress than those working in local clinics, not only because their clinical practice is generally larger than that of a local clinic physician, but also because they always have to spend extra work hours for morning meetings and academic research, and many medical center physicians have to teach residents, interns, and medical school students [7]. One study [6] reported that approximately 10% of the physicians practicing at a medical center have high-strain jobs, which normally translate in a significantly lower quality of life, especially on the psychological scale, compared with the low-strain group. Another study [24] reported an inverse relationship between the level of job stress and quality of life for physicians at primary health care centers across the nation. Despite the negative impact of the working environment, physicians who work at medical centers and have high-stress specialties did not have a significantly greater risk of having an AMI. In contrast, they had a significantly lower risk. We hypothesize that the reasons are the same: medical center physicians are not necessarily healthier but are merely more medically aware and have easier access to medical care.

There are several limitations to this study. First, because the diagnoses relied on NHIRD claims data and ICD-9-CM codes, they might have been incorrect. However, NHIRD ICD-9-CM codes have a high sensitivity and specificity for the correct diagnoses and have been validated in many studies [25–27]. Second, there was no information on the severity of AMI, working hours, or rotating night shifts; therefore, we were unable to evaluate these factors between physicians and controls. Third, we investigated only a five-year period (2007–2011), which may not be enough. Additional studies of longer periods may be needed. Finally, despite our study being nationwide, our findings may not be generalizable to other nations.

## 5. Conclusions

This study showed that significantly more physicians have chronic comorbid HTN and hyperlipidemia but a significantly lower risk for having an AMI than does the general population. There was no difference between specialties. Physicians working in medical centers have a significantly lower risk for having an AMI than do physicians working in local hospitals and local clinics. Our findings suggest that physicians are not necessary healthier but merely that they are more aware of disease and have easier access to medical care, especially if they work in a medical center, and are in a position to seek more rapid medical care to prevent catastrophic events like an AMI.

## Figures and Tables

**Table 1:** Demographic characteristics and comorbidities for physicians and controls.

Characteristic	Physicians *n* = 28,062	Controls *n* = 84,186	*P* value
Age (years)			>0.999
0–34	3,583 (12.77)	10,749 (12.77)	
35–49	14,242 (50.75)	42,726 (50.75)	
≥50	10,237 (36.48)	30,711 (36.48)	
Age (years)	46.81 ± 10.75	46.81 ± 10.75	>0.999
Gender			>0.999
Male	24,054 (85.72)	72,162 (85.72)	
Female	4,008 (14.28)	12,024 (14.28)	
Comorbidity			
DM			<0.0001
Yes	2,269 (8.09)	8,010 (9.51)	
No	25,793 (91.91)	76,176 (90.49)	
HTN			<0.0001
Yes	6,619 (23.59)	16,050 (19.06)	
No	21,443 (76.41)	68,136 (80.94)	
Hyperlipidemia			<0.0001
Yes	5,994 (21.36)	10,887 (12.93)	
No	22,068 (78.64)	73,299 (87.07)	
Geographical area			<0.0001
North	13,149 (46.86)	43,306 (51.50)	
Central	5,565 (19.83)	14,836 (17.64)	
South	8,611 (30.69)	24,165 (28.74)	
East	737 (2.63)	1,778 (2.11)	
Level of hospital employed in			
Medical Center	12,252 (43.66)		
Regional hospital	3,725 (13.27)		
Local hospital	6,382 (22.74)		
Local clinic	5,703 (20.32)		
Specialty			
Internal medicine	6,745 (24.04)		
Surgery	4,429 (15.78)		
Obstetrics and gynecology	2,251 (8.02)		
Pediatrics	3,032 (10.80)		
Emergency medicine	552 (1.97)		
Others	11,053 (39.39)		

Data are presented as *n* (%) or mean ± standard deviation. DM: diabetes mellitus; HTN: hypertension. Comparison between the two groups was evaluated using Student's *t*-test for continuous variables and Pearson *χ* ^2^ tests for categorical variables. Significance was set at *P* < 0.05 (two-tailed).

**Table 2:** The risk for an AMI between physicians and controls (conditional logistical regression analysis).

Group	Number (%)	Crude OR (95% CI)	*P* value	Adjusted OR (95% CI)	*P* value
Physicians (*n* = 28,062)	104 (0.37)	0.76 (0.61–0.94)	0.0110	0.57 (0.46–0.72)	<0.0001
Controls (*n* = 84,186)	412 (0.49)	1.000		1.000	

Adjusted by DM, HTN, hyperlipidemia, and geographical area. AMI: acute myocardial infarction; OR: odds ratio; CI: confidence interval; DM: diabetes mellitus; HTN: hypertension.

**Table 3:** The risk for an AMI between physician subgroups (conditional logistical regression analysis).

Variable	Number (%)^†^	Adjusted OR (95% CI)
Specialty		
Internal medicine	26 (0.39)	1.09 (0.65–1.83)
Surgery	29 (0.65)	1.46 (0.88–2.42)
Obstetrics and gynecology	8 (0.36)	0.70 (0.32–1.53)
Pediatrics	6 (0.20)	0.67 (0.28–1.60)
Emergency medicine	0	—
Others	35 (0.32)	1.000
Age (years)		
0–34	2 (0.06)	1.000
35–49	22 (0.15)	0.87 (0.20–3.78)
≥50	80 (0.78)	1.58 (0.37–6.77)
Gender		
Male	104 (0.43)	—
Female	0	—
Comorbidity		
DM		
Yes	39 (1.72)	1.83 (1.19–2.81)^*^
No	65 (0.25)	1.000
HTN		
Yes	87 (1.31)	7.10 (4.05–12.45)^*^
No	17 (0.08)	1.000
Hyperlipidemia		
Yes	68 (1.13)	2.69 (1.75–4.15)^*^
No	36 (0.16)	1.000
Geographical area		
North	49 (0.37)	1.000
Central	19 (0.34)	0.86 (0.50–1.47)
South	33 (0.38)	1.01 (0.65–1.58)
East	3 (0.41)	1.09 (0.34–3.56)
Level of hospital employed in		
Medical Center	9 (0.16)	0.42 (0.20–0.85)^*^
Regional hospital	13 (0.20)	0.51 (0.28–0.95)^*^
Local hospital	19 (0.51)	0.81 (0.48–1.38)
Local clinic	63 (0.51)	1.000

Adjusted by age, DM, HTN, hyperlipidemia, geographical area, and level of hospital employed in. AMI: acute myocardial infarction; OR: odds ratio; CI: confidence interval; DM: diabetes mellitus; HTN: hypertension. ^*^ *P* value < 0.05. ^†^Number (%): AMI number of the subgroup physician (percentage of “AMI number of the subgroup physicians/all numbers of the subgroup physicians”).
